# Association between cognitive functioning and microbiota-gut-brain axis mediators in a memory clinic population

**DOI:** 10.3389/fncel.2025.1550333

**Published:** 2025-03-12

**Authors:** Claudio Singh Solorzano, Cristina Festari, Peppino Mirabelli, Elisa Mombelli, Luigi Coppola, Delia Luongo, Daniele Naviglio, Andrea Soricelli, Giulia Quattrini, Marco Salvatore, Michela Pievani, Annamaria Cattaneo, Giovanni B. Frisoni, Moira Marizzoni

**Affiliations:** ^1^Laboratory of Neuroimaging and Alzheimer’s Epidemiology, IRCCS Istituto Centro San Giovanni di Dio Fatebenefratelli, Brescia, Italy; ^2^AORN Santobono-Pausilipon, UOS Laboratori di Ricerca e Biobanca, Naples, Italy; ^3^Biological Psychiatry Unit, IRCCS Istituto Centro San Giovanni di Dio Fatebenefratelli, Brescia, Italy; ^4^IRCCS SYNLAB SDN, Naples, Italy; ^5^Istituto Di Biostrutture E Bioimmagini (I.B.B.) - CNR, Naples, Italy; ^6^Department of Chemical Sciences, University of Naples Federico II, Naples, Italy; ^7^Department of Medical, Movement and Wellbeing Sciences, University of Naples Parthenope, Naples, Italy; ^8^Department of Pharmacological and Biomolecular Sciences, University of Milan, Milan, Italy; ^9^Memory Centre, Division of Geriatrics and Rehabilitation, University Hospitals of Geneva, Geneva, Switzerland; ^10^Laboratory of Neuroimaging of Aging (LANVIE), University of Geneva, Geneva, Switzerland

**Keywords:** dementia, Alzheimer’s disease, gut microbiota, microbiota-gut-brain axis, cognitive function

## Abstract

**Introduction:**

A growing body of evidence recognises the role of signaling molecule of the microbiota-gut-brain axis (MGBA) in cognitive impairment (CI), but data on the link with alterations in specific cognitive domains are limited. We compared the functioning in several cognitive domains (i.e., memory, visuo-constructional, executive, and language) among cognitively unimpaired (CU) subjects, patients with CI due to Alzheimer’s disease (CI-AD) and not due to AD (CI-NAD). Then, we investigated the association of these cognitive domains with the gut microbiota (GM), MGBA mediators, and neurodegeneration-related markers.

**Materials and methods:**

The study included 34 CI-AD, 38 CI-NAD, and 13 CU. Memory, visuo-constructional, executive, and language domains were assessed using composite measures. Faecal GM composition was inferred using 16S rRNA gene sequencing. MGBA mediators included the blood quantification of bacterial products (lipolysaccharide, LPS), cell adhesion molecules indicative of endothelial damage, vascular changes or overexpressed in response to infections, and pro- and anti-inflammatory cytokines. Neurodegeneration-related markers included plasma phosphorylated tau (p-tau181), neurofilament light chain (NfL), and glial fibrillary protein (GFAP).

**Results:**

The CI-NAD and CI-AD groups had significantly lower scores than the CU group for all cognitive domains (*p* < 0.043). Associations of MGBA modulators with cognitive functioning included pro-inflammatory cytokines, markers of endothelial dysfunction or overexpressed in response to infection in both groups of patients (|*ρ*| > 0.33, *p*s < 0.042). In the CU and CI-AD pooled group, lower cognitive functioning was specifically associated with higher abundance of *Dialister* and *Clostridia_UCG-014*, higher levels of LPS and with all neurodegeneration markers (|ρ| > 0.32, *p* < 0.048 for all). In the CU and CI-NAD pooled group, lower cognitive performance was associated with lower abundance of *Acetonema*, higher abundance of *Bifidobacterium*, *[Eubacterium]_coprostanoligenes_group* and *Collinsella*, and higher levels of vascular changes (|ρ| > 0.30, *p* < 0.049).

**Discussion:**

These results support the hypothesis that gut dysbiosis and MGBA mediators may have distinct effects on cognitive functioning and different mechanisms of action depending on the disease.

## 1 Introduction

The gut microbiota (GM) includes the complex ecosystem of bacteria, fungi, archaea, and protozoa that populates the gut (Hou et al., 2022). The GM could influence brain function and behavior through the microbiota-gut-brain axis (MGBA) (Cryan et al., 2019; Morais et al., 2021) in both physiological and pathological conditions (Cryan et al., 2020; Escobar et al., 2022). A growing body of preclinical and clinical studies recognises the role of the signaling molecule of the MGBA in mediating the association between GM dysbiosis and cognitive impairment (CI) in several neurocognitive disorders, including the Alzheimer’s disease (AD). Indeed, it has been suggested that GM dysbiosis might promote the imbalance of bacteria metabolites (MahmoudianDehkordi et al., 2019) and local inflammation (Grabrucker et al., 2023) in AD patients. These conditions have been linked to increased intestinal permeability (Pei et al., 2023), increased passage of bacteria components and cytokines into the bloodstream, and systemic inflammation (Cryan et al., 2019; Kowalski and Mulak, 2019). These processes might contribute to the alteration of the blood–brain barrier, neuroinflammation, and to the accumulation of toxic proteins in the brain (Erny et al., 2015), ultimately leading to the loss of neurons and CI (Marizzoni et al., 2017; Liu et al., 2024; Yang et al., 2024). In line, recent clinical studies in AD found a relationship between alterations of MGBA mediators (e.g., increased lipopolysaccharide and inflammatory mediators), endothelial integrity reduction, brain pathology and CI (Verhaar et al., 2022; Zhang et al., 2022; Chen et al., 2023a,b; Marizzoni et al., 2023). Similarly, several studies in healthy middle-aged and elderly subjects found that microbial community composition may be associated with cognitive performance (Canipe et al., 2021; Haimov et al., 2022; Meyer et al., 2022). However, to our knowledge, no studies described the simultaneous assessment of GM and a large panel of MGBA mediators in association with specific cognitive domains in a memory clinic population. A better understanding of the impact of MGBA on cognitive performance could help to identify potential mechanisms and new therapeutic targets for the treatment and prevention of CI.

In this cross-sectional study, we explored the association between specific cognitive domains (i.e., memory, visuo-constructional, executive, and language) with faecal bacterial genera, MGBA mediators, and neurodegenerative-related markers in older adults with normal cognition (CU), patients with CI due to AD (CI-AD), and patient with CI not due to AD (CI-NAD). Our main hypothesis is that the CI-AD and CI-NAD groups showed specific MGBA mediator profiles related to the altered cognitive domains.

## 2 Materials and methods

Several aspects of the subjects, study design and data analyses used in the present study have been described elsewhere (Marizzoni et al., 2023). Here, we added the assessment of cognitive functioning domains in relationship with GM and a wide range of MGBA mediators, and we included the quantification of plasma levels of GFAP, considered a proxy for neuroinflammation (Abdelhak et al., 2022) and recently recognized as biomarker of AD pathophysiology (Jack et al., 2024).

### 2.1 Participants

Participants (*n* = 85) were selected from a large Italian study on amyloid imaging, the Incremental Diagnostic Value of [18F]florbetapir Amyloid Imaging [INDIA-FBP] study (Boccardi et al., 2016) and underwent a multi-domain neuropsychological evaluation, a [18F]florbetapir PET, and blood and stool exams. Inclusion criteria were age between 50 and 85 years, availability of an informant (spouse, adult child, or another knowledgeable informant), and being native/fluent Italian speakers to complete the neuropsychological tests correctly. Exclusion criteria included being under antibiotic and anti-inflammatory treatment over the past 3 months or a past diagnosis of major depression or any other psychiatric disorders.

The neuropsychological evaluation covered four cognitive domains and related tests: (i) memory [Story Recall Test, total immediate and delayed recall of the Rey Auditory Verbal Learning Task (RAVLT)] (Novelli et al., 1986a; Carlesimo et al., 1996); (ii) visuo-constructional ability (Rey-Osterrieth Complex Figure) (Caffarra et al., 2002); (iii) executive functions [Trail Making Test part A (TMT-A), Raven progressive matrices] (Giovagnoli et al., 1996; Amodio et al., 2002; Caffarra et al., 2003); (iv) language (Token Test, phonemic and semantic verbal fluency test) (Novelli et al., 1986b; Spinnler and Tognoni, 1987). Participants were classified as CU (i.e., no more than one neuropsychological test was abnormal) and CI (i.e., two or more neuropsychological tests were beyond the normal range). Some participants could not complete specific neuropsychological tests due to the severity of cognitive impairment. Composite scores for each cognitive domain could not be calculated for these individuals, leading to missing data in the analysis including the cognitive domains. The [18F]florbetapir standardized uptake value ratio (SUVr) was computed as the ratio of the global cortical (frontal, parietal, temporal, anterior cingulate, posterior cingulate, and precuneus) to the cerebellar uptake. Participants were also classified as amyloid-positive (CI-AD) and amyloid-negative (CI-NAD and CU) based on an established cut-off (SUVr>1.10) (Clark et al., 2012).

Written informed consent was obtained from all participants and covered sample processing and analyses. The study was approved by the Ethics Committee of the IRCCS Fatebenefratelli (approval date November 18, 2014, number 57/2014) and was conducted according to the Declaration of Helsinki.

### 2.2 Faecal bacterial composition

Stool samples were collected in sterile plastic cups from participants at their own home, stored at −20°C, and delivered within 24 h to the IRCCS Fatebenefratelli Institute in Brescia, where they were stored at −20°C until processing.

Faecal microbiota analyses were performed as reported elsewhere (Marizzoni et al., 2023). Briefly, faecal DNA was extracted using the QIAamp Fast DNA Stool Mini Kit (Qiagen Retsch GmbH, Hannover, Germany), the V3-V4 regions of the bacterial 16S rRNA gene were amplified by Illumina’s 16S Metagenomic Sequencing Library Preparation protocol and sequenced on Illumina MiSeq platform. The raw 16S data were processed using QIIME2 (Bolyen et al., 2019) and underwent the denoising process using DADA2 (Callahan et al., 2016). The SILVA reference 16S rRNA gene database 138 was used to assign taxonomies (Quast et al., 2012).

### 2.3 Microbiota-gut-brain axis mediators

A series of markers were selected to investigate various potential metabolic, endothelial, and immune mediators of MGBA, and measured in plasma by ELISA (Pierce LAL Chromogenic Endotoxin Quantitation Kit, Thermo Fisher Scientific): (i) lipopolysaccharide (LPS), an important microbial-generated neurotoxin (Zhao et al., 2019), (ii) sVCAM-1 and sPECAM-1, as endothelial damage markers (Chen et al., 2023a,b; Sim et al., 2024), (iii) sP-Selectin, as an indicator of vascular damage (Zuliani et al., 2008), (iv) sICAM3 and sCD44, as markers of immune response to infection (Baaten et al., 2010; Guerra-Espinosa et al., 2024).

A panel of cytokines typically altered in AD (i.e., IL1β, TNFα, IL-18, IL-10) was measured using semi-quantitative real-time PCR (Brosseron et al., 2018). The total RNA isolation was performed using the PAXgene blood miRNA kit and according to the manufacturer’s protocol (PreAnalytiX, Hombrechtikon, CHE). Each target gene was normalized to the geometric mean of the expression of three reference genes (i.e., glyceraldehyde-3-phosphate dehydrogenase, beta-actin, and beta-2-microglobulin) using the TaqMan assays on a 384-well Real-Time PCR system (Biorad Laboratories, Hercules, USA). The relative target gene expression of each gene in patients compared to controls was determined using the Pfaffl method (Pfaffl, 2001).

### 2.4 Neurodegeneration-related markers

The SUVrs were calculated as global measures (Marizzoni et al., 2020). Venous blood samples were collected from all participants using a 4 mL K3-ethylenediaminetetraacetic acid (EDTA) vacutainer, and centrifuged within two hours of collection at 3400 g for 10 min at 4°C to obtain plasma. Plasma samples were then aliquoted and stored at −80°C until testing.

Plasma concentrations of neurofilament light chain (NfL) (NF-Light immunoassay Advantage kit; Cat. No. 103400), glial fibrillary acidic protein (GFAP) (GFAP Human Discovery Kit; Cat. No. 102336), and p-tau181 (p-tau181 V2 Advantage Kit; Cat. No. 103714) were measured using the ultrasensitive Simoa SR-X instrument following the manufacturer’s recommended protocol obtained by Quanterix, Billerica, USA.

### 2.5 Statistical analysis

Statistical analyses were performed using Rstudio version 4.4.1. We computed composite scores for each cognitive domain (i.e., memory, visuo-constructional, executive, and language). Raw scores on each test were *z*-transformed according to the performance distribution of the entire sample. Then, *z*-scores for each test were averaged for each domain. Higher scores mean better functioning in the specific domain. For this purpose, we computed the reverse score of the TMT-A to associate higher scores with a better performance on the test.

The normal distribution of the variables was determined by the Shapiro–Wilk test. Descriptive statistics for the total sample and the three groups of interest (i.e., CU, CI-AD, CI-NAD) were reported as mean (M) and standard deviation (SD) for continuous variables and as number of participants (N) and percentage (%) for categorical variables. The ANOVA or Kruskall-Wallis test, according to data distribution with Bonferroni corrections for multiple comparisons, were used to compare continuous variables. The Pearson chi-square test was used to compare categorical variables. For graphical purposes, GM and putative MGBA mediators were reported as percentage differences of the CI groups (i.e., CI-NAD or CI-AD) versus CU.

Partial Spearman’s rank correlation was used to test the association between cognitive domains with genera, MGBA mediators, and neurodegeneration-related markers, controlling for the effect of age. Partial correlations were performed for the CU and CI-NAD pooled group and for the CU and CI-AD pooled group. The significance was set at *p* < 0.050 (two-tailed). For hypothesis validation analyses, associations were selected if their Spearman’s *rho* value was>0.4 to include only those with at least moderate association (Prion and Haerling, 2014).

## 3 Results

Demographic and clinical characteristics were as expected for this population (Table 1 and Supplementary Table 1) Reported medications were comparable between the two patient groups. Cognitive domains.

**Table 1:** Demographic characteristics of study participants (*N* = 85).

Variable	Total sample	CU	CI-NAD	CI-AD	Comparison test^a^
	*N* = 85	*N* = 13	*N* = 38	*N* = 34	*p*-value
Age (years)	70.16 ± 6.76	69.60 ± 7.01	69.80 ± 7.39	70.77 ± 6.05	0.791
Female	44 (51.8)	7 (53.8)	21 (55.3)	16 (47.0)	0.775
Education (years)	8.62 ± 4.31	9.08 ± 5.19	8.50 ± 3.94	8.69 ± 4.48	0.989
BMI (kg/cm^2^)	25.11 ± 3.34	24.98 ± 3.44	25.31 ± 3.78	24.93 ± 2.82	0.839
APOEe4 carrier status^b^	25 (32.5)	2 (18.1)	3 (8.8)	20 (64.5)	< 0.001
MMSE	24.28 ± 4.63	28.31 ± 1.11	24.76 ± 3.88	22.21 ± 5.12	< 0.001
Clinical stage					
MCI	43 (50.6)	-	21 (55.3)	22 (64.7)	0.415
Dementia	29 (34.1)	-	17 (44.7)	12 (35.3)	0.415
Drugs used					
AChE inhibitors	5 (5.8)	-	2 (5.3)	3 (8.8)	0.553
Memantine	1 (1.2)	-	1 (2.6)	0 (0)	0.953
Antidepressant/hypnotic/anxiolytic	29 (34.1)	-	13 (34.2)	16 (47.0)	0.267
Antipsychotic	4 (4.7)	-	3 (7.9)	1 (2.9)	0.916
Nutritional supplements	11 (12.9)	-	4 (10.5)	7 (20.5)	0.236
Amyloid load ([18F]florbetapir SUVr)	1.08 ± 0.21	0.95 ± 0.07	0.92 ± 0.09	1.30 ± 0.13	< 0.001

Figure denotes mean (SD) and number (%). CU: cognitively unimpaired persons; CI-NAD: patients with cognitive impairment not due to AD; CI-AD: patients with cognitive impairment due to AD; BMI: body mass index; MMSE: Mini-Mental State Examination; AchE inhibitors, acetylcholinesterase inhibitors; SUVr, standardized uptake value ratio.

^a^ Statistical difference among the three groups (i.e., CU, CI-NAD, and CI-AD) using ANOVA or Kruskal-Wallis for continuous variables and Chi-squared test for categorical variables.

^b Missing data for 8 participants: 2 in CU, 4 in CI-NAD, and 3 in CI-AD.^

**Figure 1:**
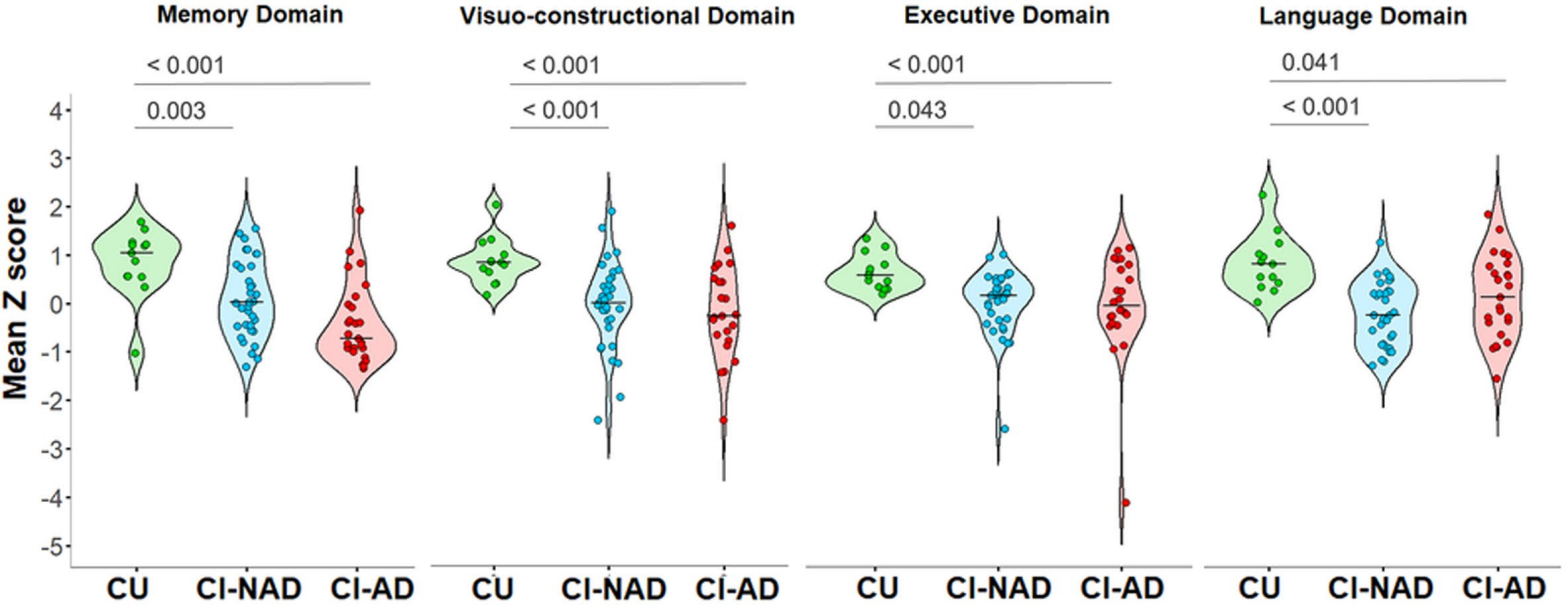
Violin plot of the distribution of cognitive domains’ *z* scores for the three groups (CU, CI-NAD, CI-AD). The plots show the median (indicated by the black horizontal band), the first through the third interquartile range (the vertical band), and an estimator of the density (thin vertical curves) of each cognitive domain functioning in each group. The reported *p*-values were calculated by using one-way ANOVA with Bonferroni’s correction for normal distributed variables (i.e., visuo-constructional domain) or Kruskall-Wallis test with Bonferroni’s correction for non-normal distributed variables (i.e., memory domain, executive domain, and language domain). CI-AD, patients with cognitive impairment due to AD; CI-NAD, patients with cognitive impairment not due to AD; CU, cognitively unimpaired persons.

All domains showed significant differences between groups memory domain: *H* (2) = 20.373, *p* < 0.001, *n* = 84; visuo-constructional domain: *F*(2,70) = 7.942, *p* < 0.001, *n* = 73; executive domain: *H* (2) = 14.75, *p* < 0.001, *n* = 84; language domain: *H* (2) = 15.049, *p* < 0.001, *n* = 80. In particular, the CI-NAD and CI-AD groups had significantly lower scores than the CU group for all domains (Figure 1). However, no significant differences were found when comparing CI-NAD and CI-AD (Figure 1).

### 3.1 GM, microbiota-gut-brain axis mediators and neurodegeneration-related markers

**Figure 2:**
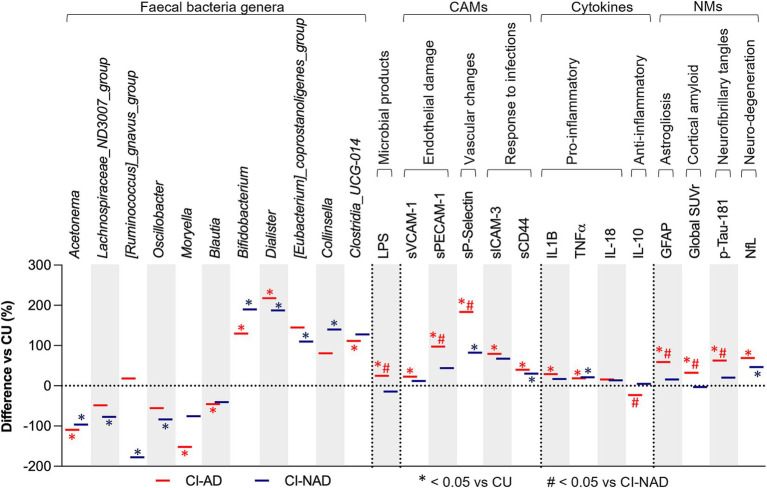
GM and MGBA putative mediators of study participants. Bars denote percentage difference in CI-AD and CI-NAD patients versus unimpaired control subjects (CU). The percentage difference has been calculated using control subjects as reference (represented by the threshold line at 0). *p*-values were calculated by using one-way ANOVA or Kruskall-Wallis test (accordingly with data distribution) with Bonferroni’s test for multiple comparison correction on raw data. Statistical significances is represented by * at *p* < 0.05, *** at *p* < 0.001 comparing CI-AD and CI-NAD versus CU and by # at *p* < 0.05 comparing CI-AD versus CI-NAD. CI-AD, patients with cognitive impairment due to AD; CI-NAD, patients with cognitive impairment not due to AD; CU = cognitively unimpaired persons.

When focusing on GM composition, both CI groups showed a lower abundance of *Acetonema* (−96.3% in CI-NAD, −109.5% in CI-AD; Figure 2) and a higher abundance of *Bifidobacterium* (+187.2% in CI-NAD, +125.3% in CI-AD) and *Dialister* (+187.1% in CI-NAD, +217.7% in CI-AD) compared to CU. Similarly, when considering MGBA mediators and neurodegenerative markers, higher levels of sP-Selectin (+82.2% in CI-NAD, +183.4% in CI-AD), sCD44 (+30.3% in CI-NAD, +39.8% in CI-AD), TNFα (+21.1% in CI-NAD, +18.7% in CI-AD), and NfL (+46.6% in CI-NAD, +69.1% in CI-AD) were reported in both CI groups compared to CU.

The CI-AD group was characterized by a decreased abundance of *Moryella* (−151.3%), *Blautia* (−45.5%), and an increased abundance of *Clostridia_UCG-014* (+111.6) compared to CU. The CI-NAD showed a lower abundance of *Lachnospiraceae_ND3007_group* (−77.2%), *[Ruminococcus]_gnavus_group* (−177.5%), and *Oscillobacter* (−83.5%), and a higher abundance of *Collinsella* (139.6%) compared to CU. On the other hand, CI-AD group was characterized by higher levels of LPS (+24.7%), sVCAM-1 (+22.7% *vs* CU), sPECAM1 (+97.4% *vs* CU), sICAM-3 (+97.2% *vs* CU), and the pro-inflammatory cytokine IL-1β (+28.9% vs. CU), and lower levels of the anti-inflammatory cytokine IL-10 (−22.9% vs. CI-NAD). Significantly greater levels of plasma GFAP (+59.1% vs. CU), amyloid (+32.2% vs. CU), and p-tau181 (+62.6% vs. CU) were found for the CI-AD when compared to CU.

### 3.2 Association between cognitive domains with microbial genera, MGBA and neurodegenerative-related markers

**Figure 3:**
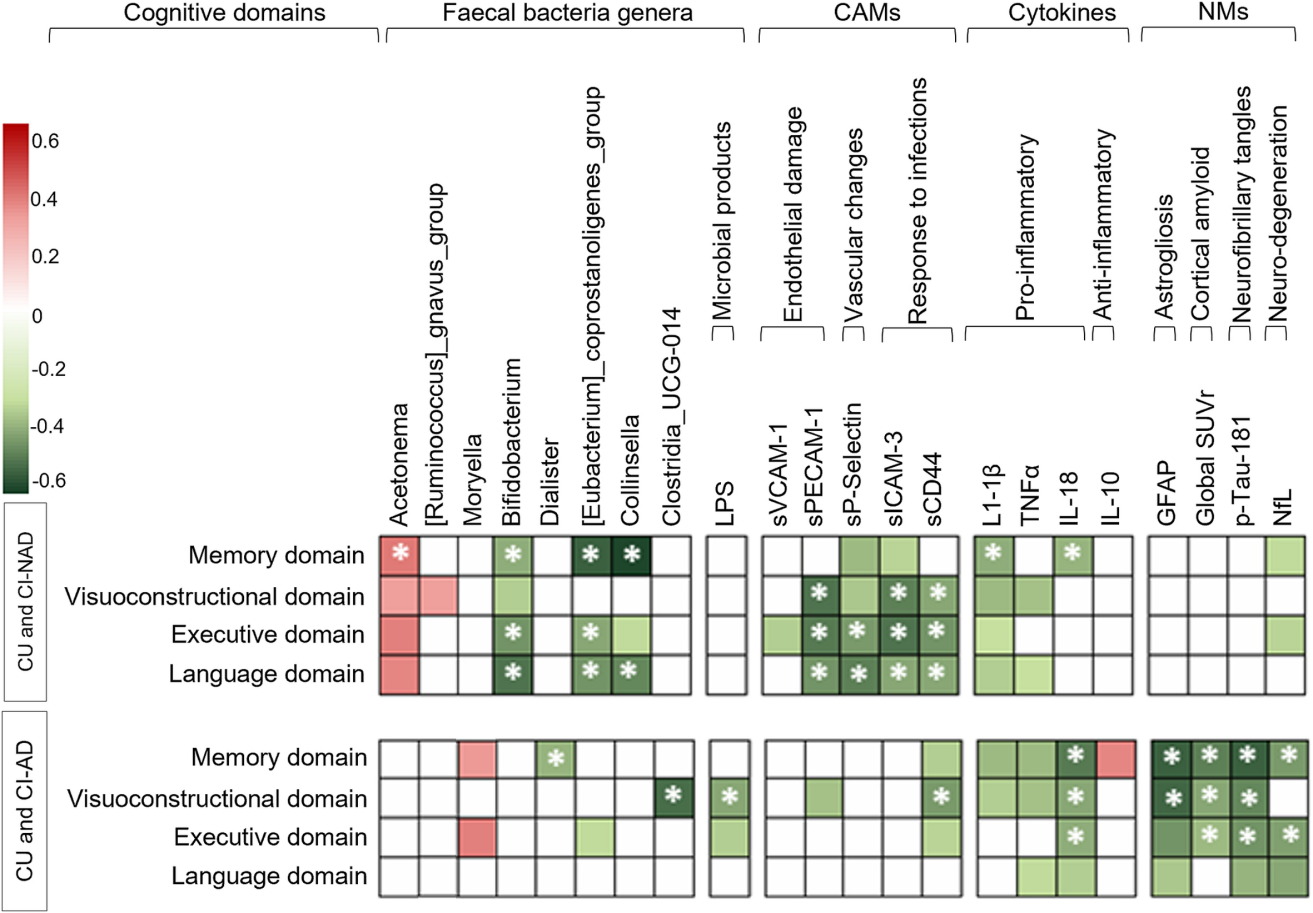
Heatmap of the Spearman’s rho coefficient values (red: positive; green: negative) indicating significant age-adjusted association in CU and CI-NAD or CU and CI-AD (*p* < 0.05, for exact p-values and confidence intervals please refer to Supplementary Table 2). Asterisks indicate moderate associations (*ρ* > 0.4). For cognition domains, higher values reflected better cognitive performance. CAMs, cell adhesion molecules; CI-AD, patients with cognitive impairment due to AD; CI-NAD, patients with cognitive impairment not due to AD; CU, cognitively unimpaired persons; NMs, neurodegenerative-related markers.

Figure 3 shows the association of cognitive domains with microbial genera, MGBA, and neurodegenerative-related markers. In both patient groups, lower levels of pro-inflammatory cytokines, CAMs indicative of endothelial damage or upregulated immune response, and NfL were associated with better cognitive performances (|*ρ*| > 0.33, *p*s < 0.042). GM alteration was directly associated with cognitive performance, but the genera involved were different in CI-AD and CI-NAD (Supplementary Table 2). Furthermore, lower levels of LPS, amyloid, p-tau181, GFAP, and high expression of IL-10 were associated with better cognitive performance only in the CU and CI-AD pooled group (|*ρ*| > 0.34, *p*s < 0.037).

**Figure 4:**
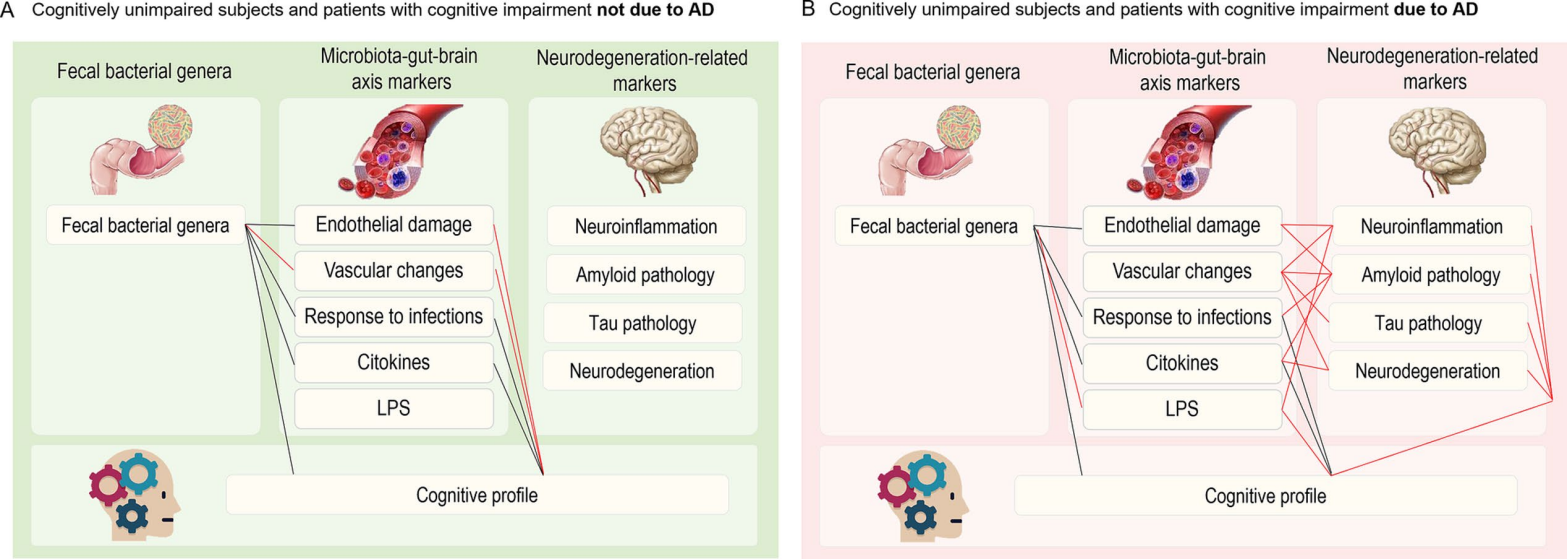
Closest associations (*ρ* > 0.4) between faecal bacterial genera, microbiota-gut-brain axis mediators, neurodegeneration-related markers and cognitive profile in CU and CI-NAD **(A)** and CU and CI-AD **(B)**. Black lines indicate common paths for both groups, whereas red lines indicate paths specific to CI-NAD or CI-AD.

Figure 4 refines the previously published model (Marizzoni et al., 2023) on the possible relationships between GM, MGBA mediators, and neurodegeneration-related markers in the CI-AD (panel A) and CI-NAD (panel B) groups. Overall, alterations in the GM composition and inflammatory profile (increased expression of cytokines and upregulation of CAMs) were associated with greater cognitive impairment in both CI-NAD and CI-AD. Interestingly, direct and group-specific associations between MGBA mediators and CI involved endothelial and vascular damage markers in CI-NAD and LPS in CI-AD. Furthermore, MGBA mediators were widely associated with neurodegeneration-related markers, and the latter were associated with CI only in CI-AD.

## 4 Discussion

This cross-sectional study primarily investigated the association of cognitive domains (i.e., memory, visuo-constructional, executive, and language) with faecal bacterial genera, MGBA mediators, and neurodegenerative-related markers in a cohort of cognitively unimpaired persons, patients with cognitive impairment due to AD, and patients with cognitive impairment not due to AD. Our principal results revealed the presence of an extensive association of GM and MGBA mediators with cognitive impairment, some common to both patient groups, and some specific to CI-AD or CI-NAD.

Common associations included pro-inflammatory cytokines, soluble CAMs involved in endothelial damage or overexpressed in response to infection, and neurodegeneration markers. These results confirmed previous human findings showing that levels of pro-inflammatory cytokines (i.e., IL-1β, TNFα, and IL-18), sCAMs (i.e., sVCAM1, sPECAM1, sICAM3, sCD44), and NfL were associated with the progression of cognitive decline in dementia (Nielsen et al., 2007; Leblhuber et al., 2015; Ashton et al., 2021; Drake et al., 2021; Rasi Marzabadi et al., 2021; Hosoki et al., 2023). The increase in sCAMs is strictly related with systemic inflammation and the disruption of the blood–brain barrier’s integrity, which ultimately might lead to neuroinflammation and loss of neurons (Cryan et al., 2019; Liu et al., 2020; Loh et al., 2024). Therefore, these findings support the crucial role of systemic inflammation in influencing brain functioning and contributing to the development of cognitive impairment.

Specific associations of MGBA modulators with cognitive functioning for the CI-AD group included the abundance of *Dialister* and *Clostridia_UCG-014,* as well as the levels of LPS and IL-10 expression. Accumulating evidence supports a close connection between GM dysbiosis and AD, although studies disagree on the identity of the genera involved (Cattaneo et al., 2017; Vogt et al., 2017; Guo et al., 2021; Ling et al., 2021; Laske et al., 2022). A few studies showed that the *Dialister* genus and Clostridia class could be related to pathological mechanisms in AD (Vogt et al., 2017; Ling et al., 2021; Kaiyrlykyzy et al., 2022; Khedr et al., 2022). In line, we found a specific association between the higher abundance of *Dialister* and *Clostridia_UCG-014* genera and greater impairment of memory, executive, and language cognitive domains in the CI-AD group. In our study, higher LPS levels were related to poor visuo-constructional and executive cognitive functioning, following the evidence of a strict relationship between LPS and the progressive cognitive decline associated with AD (Zhan et al., 2018; Ghosh et al., 2020). Thus, these findings support the endotoxin hypothesis of AD (Brown and Heneka, 2024) and the evidence of higher levels of LPS in AD patients compared to CU (Zhang et al., 2009; Andreadou et al., 2021). The relationship between IL-10 and the memory domain aligns with the neuroprotective role of anti-inflammatory processes in AD (Su et al., 2016; Porro et al., 2020). Some evidence reported that high expression of IL-10 was associated with low brain amyloid load in humans (D’Anna et al., 2017; Marizzoni et al., 2020) and with neurogenesis processes and enhanced cognition in animal models of AD (Kiyota et al., 2012; Guillot-Sestier et al., 2015).

Specific associations of MGBA modulators with cognitive functioning for the CI-NAD group included the abundance of *Acetonema*, *Bifidobacterium*, *[Eubacterium]_coprostanoligenes_group*, *Collinsella*, and sP-Selectin levels. These genera have been associated with cognitive decline but with conflicting results (Li et al., 2019; Rueda-Ruzafa et al., 2019; Nishiwaki et al., 2022). In particular, we found specific associations between the high abundance of *Bifidobacterium*, *[Eubacterium]_coprostanoligenes_group*, *Collinsella* genera and greater impairment of memory, executive, and language cognitive domains; on the other side, the high abundance of *Acetonema* could be a protective factor for cognitive functioning. Moreover, vascular damage and platelet activation markers (i.e., sP-Selectin) were strictly associated with cognitive impairment in all the evaluated domains, suggesting a potentially high number of vascular dementias in the CI-NAD group (Toth et al., 2017; van der Flier et al., 2018; Morgan and Mc Auley, 2024).

Concerning the neurodegeneration-related biomarkers, neuroinflammation and tau pathology play a significant role in the definition of cognitive impairment only in the CI-AD group. Beyond the expected association between ATN cascade markers and cognitive impairment (Hanseeuw et al., 2019), astrogliosis, as reflected by the increase in GFAP levels, seems to play a role in the cognitive profile of AD patients. The association between increased levels of GFAP and higher cognitive impairment in the AD group are in line with recent studies that posited specific reactive astrogliosis in AD and its correlation with the severity of cognitive impairment (Bettcher et al., 2021; Oeckl et al., 2022; Peretti et al., 2024).

These findings highlight the potential of MGBA mediators as promising biomarkers for cognitive impairment (Cryan et al., 2019; Morais et al., 2021). The possibility to integrate MGBA variables with neuroimaging and genetic markers for AD diagnosis or for differentiating AD-related and non-AD-related cognitive impairment needs further investigation. Moreover, future research should prioritize longitudinal studies to validate the association of MGBA mediators with cognitive decline. Mechanistic investigations are needed to unravel causal relationships between specific microbial genera, MGBA signaling molecules, and cognitive functions. Such studies could pave the way for targeted therapeutic interventions, such as microbiota-based therapies, dietary interventions, or pharmacological modulation of MGBA pathways, to mitigate or prevent cognitive decline (Li et al., 2023).

We are aware of several limitations of the study. Firstly, its cross-sectional design and the small sample size make it difficult to generalize the results and the causality of the conclusions. More independent, longitudinal, and large cohort studies are needed to confirm the present results. Secondly, other potential MGBA mediators that could play an important role in neurodegenerative diseases (e.g., short-chain fatty acids, neurotransmitters, estrogens, etc.) (Mayer et al., 2022) were not considered. Third, we did not include the severity and duration of dementia as covariates in the models. The progression of cognitive decline should be considered in future studies to identify better the timing and the most important players involved in the different phases of the disease. Fourth, the CI-NAD group is not characterized by a specific neurodegenerative diagnosis. However, this could represent a starting point for future works with well-characterized forms of non-AD dementias (e.g., vascular cognitive impairment, frontotemporal lobar degeneration) better to outline the cognitive impairment profile of non-AD-related neurodegenerative disorders. Finally, it should be noted that studies addressing changes in the whole GM in AD often report conflicting results due to the use of different methods for extraction, sequencing, and analyses of the microbiome profile. A harmonization procedure is needed to move the field forward.

In conclusion, these results suggest that gut microbiota and MGBA mediators in cognitive impairment may have distinct effects and mechanisms of action depending on the disease. In the CI-NAD group, MGBA mediators – particularly endothelial damage and vascular changes – are directly associated with cognitive impairment. In the CI-AD group, the effect of the MGBA mediators on cognition seemed associated with the modulation of the central neurodegeneration-related markers (i.e., GFAP, cortical amyloid, and p-tau181). MGBA variables are promising markers for cognitive impairment monitoring and treatment and deserve further investigations.

## Data Availability

The datasets presented in this study can be found in online repositories. The names of the repository/repositories and accession number(s) can be found at: https://www.ebi.ac.uk/ena, PRJEB55056.
