# Supplementary material for: Association between cognitive functioning and microbiota-gut-brain axis mediators in a memory clinic population
Source: Front Cell Neurosci. 2025 Mar 12;19:1550333. doi: 10.3389/fncel.2025.1550333 (PMC11936893; doi:10.3389/fncel.2025.1550333)
Supplement: Supplementary file 1 [file Table_1.docx]

**Supplementary Table 1.** Risk factors of study participants.

**Supplementary Table 2.** Spearman’s *rho* coefficient values with exact *p*-values and confidence intervals.

**Supplementary Method 1.** DNA amplification, barcoding and sequencing.

**Supplementary Table 1.** Risk factors of study participants.

|  | ***CU*** | ***CI-NAD*** | ***CI-AD*** | ***p -value*** |
| --- | --- | --- | --- | --- |
| ***N*** | 13 | 38 | 34 |  |
| Hypertension | 4 (31) | 26 (68) | 16 (47) | **.036**^a^ |
| Diabetes | 2 (15) | 6 (16) | 4 (12) | .878 |
| Vascular disease | 4 (31) | 11 (29) | 4 (12) | .159 |
| Hypercholesterolemia | 7 (54) | 18 (47) | 15 (44) | .835 |
| Stroke | 1 (8) | 6 (16) | 6 (18) | .693 |

^a^ CU *vs* CI-NAD, *p*=.017 and CI-AD *vs* CI-NAD, *p*=.067.

Figure denotes number (%). Statistical difference between the groups was assessed by Chi-squared test.

Abbreviations: CU: cognitively unimpaired persons; CI-NAD: patients with cognitive impairment not due to AD; CI-AD: patients with cognitive impairment due to AD.

**Supplementary Table 2.** Spearman’s *rho* coefficient values with exact *p*-values and confidence intervals.

| ***Variables*** | ***rho*** | ***p-value*** | ***95% CI*** |
| --- | --- | --- | --- |
| ***CU and CI-NAD*** |  |  |  |
| *Acetonema*/memory domain | 0.413 | 0,019 | [0.082;0.622] |
| *Acetonema*/visuoconstructional domain | 0.314 | 0.048 | [0.015;0.575] |
| *Acetonema*/executive domain | 0.339 | 0.029 | [0.033;0.582] |
| *Acetonema*/language domain | 0.327 | 0.039 | [0.010;0.601] |
| *[Ruminococcus]_gnavus_group*/visuoconstructional domain | 0.323 | 0.042 | [0.017;0.617] |
| *Bifidobacterium*/memory domain | -0.422 | 0.013 | [-0.678;-0.082] |
| *Bifidobacterium*/visuoconstructional domain | -0.359 | 0.022 | [-0.607;-0.057] |
| *Bifidobacterium*/executive domain | -0.487 | 0.001 | [-0.686;-0.213] |
| *Bifidobacterium*/language domain | -0.600 | < 0.001 | [-0.747;-0.354] |
| *[Eubacterium]_coprostanoligenes_group*/memory domain | -0.574 | < 0.001 | [-0.764;-0.295] |
| *[Eubacterium]_coprostanoligenes_group*/executive domain | -0.411 | 0.014 | [-0.633;-0.047] |
| *[Eubacterium]_coprostanoligenes_group*/language domain | -0.462 | 0.002 | [-0.691;-0.155] |
| *Collinsella*/memory domain | -0.582 | < 0.001 | [-0.757;-0.340] |
| *Collinsella*/executive domain | -0.301 | 0.049 | [-0.531;-0.002] |
| *Collinsella*/language domain | -0.505 | < 0.001 | [-0.704;-0.295] |
| sVCAM-1/Executive domain | -0.458 | 0.007 | [-0.752;-0.098] |
| sPECAM-1/visuoconstructional domain | -0.536 | 0.002 | [-0.720;-0.236] |
| sPECAM-1/executive domain | -0.516 | 0.002 | [-0.749;-0.220] |
| sPECAM-1/language domain | -0.466 | 0.008 | [-0.699;-0.184] |
| sP-Selectin/memory domain | -0.328 | 0.045 | [-0.655;-0.049] |
| sP-Selectin/visuoconstructional domain | -0.384 | 0.032 | [-0.585;-0.111] |
| sP-Selectin/executive domain | -0.473 | 0.005 | [-0.681;-0.222] |
| sP-Selectin/language domain | -0.530 | 0.002 | [-0.729;-0.245] |
| sICAM-3/memory domain | -0.322 | 0.045 | [-0.648;-0.004] |
| sICAM-3/visuoconstructional domain | -0.515 | 0.002 | [-0.680;-0.251] |
| sICAM-3/executive domain | -0.516 | 0.002 | [-0.705;-0.261] |
| sICAM-3/language domain | -0.410 | 0.020 | [-0.612;-0.147] |
| sCD44/visuoconstructional domain | -0.423 | 0.010 | [-0.678;-0.093] |
| sCD44/executive domain | -0.452 | 0.004 | [-0.639;-0.173] |
| sCD44/language domain | -0.407 | 0.014 | [-0.617;-0.100] |
| L1-1β/memory domain | -0.437 | 0.005 | [-0.649;-0.147] |
| L1-1β/visuoconstructional domain | -0.399 | 0.011 | [-0.628;-0.112] |
| L1-1β/executive domain | -0.290 | 0.049 | [-0.554;-0.036] |
| L1-1β/language domain | -0.345 | 0.029 | [-0.642;-0.017] |
| TNFα/visuoconstructional domain | -0.374 | 0.017 | [-0.654;-0.064] |
| TNFα/language domain | -0.397 | 0.011 | [-0.695;-0.026] |
| IL-18/memory domain | -0.401 | 0.011 | [-0.694;-0.066] |
| NfL/memory domain | -0.297 | 0.048 | [-0.560;-0.002] |
| NfL/executive domain | -0.299 | 0.047 | [-0.601;-0.058] |
| ***CU and CI-AD*** |  |  |  |
| *Moryella*/memory domain | 0.298 | 0.049 | [0.001;0.598] |
| *Moryella*/executive domain | 0.301 | 0.043 | [0.002;0.561] |
| *Dialister*/memory domain | -0.429 | 0.010 | [-0.687;-0.091] |
| *[Eubacterium]_coprostanoligenes_group*/executive domain | -0.322 | 0.039 | [-0.597;-0.005] |
| *Clostridia_UCG-014*/visuoconstructional domain | -0.551 | < 0.001 | [-0.795;-0.211] |
| LPS/visuoconstructional domain | -0.498 | 0.007 | [-0.749;-0.154] |
| LPS/executive domain | -0.344 | 0.037 | [-0.632;-0.007] |
| sPECAM-1/visuoconstructional domain | -0.409 | 0.028 | [-0.712;-0.057] |
| sCD44/memory domain | -0.416 | 0.020 | [-0.742;-0.020] |
| sCD44/ visuoconstructional domain | -0.466 | 0.009 | [-0.720;-0.132] |
| sCD44/executive domain | -0.329 | 0.038 | [-0.651;-0.004] |
| L1-1β/memory domain | -0.426 | 0.011 | [-0.713;-0.053] |
| L1-1β/visuoconstructional domain | -0.385 | 0.032 | [-0.665;-0.029] |
| TNFα/memory domain | -0.430 | 0.009 | [-0.736;-0.094] |
| TNFα/visuoconstructional domain | -0.364 | 0.044 | [-0.652;-0.134] |
| TNFα/language domain | -0.332 | 0.048 | [-0.595;-0.003] |
| IL-18/memory domain | -0.579 | < 0.001 | [-0.785;-0.301] |
| IL-18/visuoconstructional domain | -0.518 | 0.002 | [-0.739;-0.247] |
| IL-18/executive domain | -0.475 | 0.005 | [-0.724;-0.152] |
| IL-18/language domain | -0.454 | 0.008 | [-0.745;0.076] |
| IL-10/memory domain | 0.321 | 0.048 | [0.009;0.571] |
| GFAP/memory domain | -0.576 | < 0.001 | [-0.801;0.242] |
| GFAP/visuoconstructional domain | -0.559 | 0.002 | [-0.779;-0.256] |
| GFAP/executive domain | -0.506 | 0.005 | [-0.767;-0.165] |
| GFAP/language domain | -0.316 | 0.047 | [-0.615;-0.003] |
| Global SUVr/memory domain | -0.540 | < 0.001 | [-0.767;-0.202] |
| Global SUVr/visuoconstructional domain | -0.458 | 0.005 | [-0.696;-0.147] |
| Global SUVr/executive domain | -0.301 | 0.048 | [-0.591;-0.002] |
| p-Tau-181/memory domain | -0.599 | <0.001 | [-0.763;-0.339] |
| p-Tau-181/visuoconstructional domain | -0.480 | 0.007 | [-0.722;-0.168] |
| p-Tau-181/executive domain | -0.399 | 0.026 | [-0.649;-0.073] |
| p-Tau-181/language domain | -0.333 | 0.046 | [-0.642;-0.037] |
| NfL/memory domain | -0.322 | 0.048 | [0.626;0.001] |
| NfL/executive domain | -0.375 | 0.036 | [-0.584;-0.009] |
| NfL/language domain | -0.341 | 0.047 | [-0.503;-0.004] |

**Supplementary Method 1.** DNA amplification. barcoding and sequencing.

Bacterial DNA was amplified and purified according to 16S Metagenomic Sequencing Library Preparation protocol by Illumina. This protocol allows as a first step to amplify the regions V3 and V4 of the bacterial ribosomal RNA 16S gene. by using the suggested primers (16S Amplicon PCR Forward Primer = 5'TCGTCGGCAGCGTCAGATGTGTATAAGAGACAGCCTACGGGNGGCWGCAG; 16S Amplicon PCR Reverse Primer = 5'GTCTCGTGGGCTCGGAGATGTGTATAAGAGAC AGGACTACHVGGGTATCTAATCC)

and the suggested cycling conditions (3’ at 95°C; 25 cycles: 30’’ at 95°C. 30’’ at 55°C. 30’’ at 72°C; 5’ at 72°C). The resulting amplicon DNA was immediately purified with a magnetic bead step. washed in 80% ethanol and resuspended in Tris-HCl 10 mM. Amplicon DNA integrity was assessed by 2.0% agarose gel electrophoresis on gels containing 0.8 mg/mL ethidium bromide and stored at -20°C. Within one week from storage. amplicon DNA was uniquely dual-indexed. by using the suggested indices (Nextera XT) and the suggested cycling conditions (3’ at 95°C; 8 cycles: 30’’ at 95°C. 30’’ at 55°C. 30’’ at 72°C; 5’ at 72°C). The resulting indexed DNA was immediately purified with a magnetic bead step. washed in 80% ethanol. resuspended in Tris-HCl 10 mM and stored at -20°C. Within one week from storage. indexed DNA was quantified using fluorometric quantification (Qubit. Invitrogen) and the amplicon length was determined by using a Bioanalyzer DNA 1000 chip (Agilent). DNA was then normalized to 4nM. pooled. denatured with NaOH 0.1N. diluted to the final concentration of 10pM and loaded into the MiSeq v3 cartridge (Illumina).
